# Levistilide A Promotes Expansion of Human Umbilical Cord Blood Hematopoietic Stem Cells by Enhancing Antioxidant Activity

**DOI:** 10.3389/fphar.2022.806837

**Published:** 2022-02-17

**Authors:** Mei He, Hui Xu, Guangju Liu, Ming Yang, Wenshan Zhang, Yafang Li, Hexiao Zhang, Chaoqun Wang, Yiran Zhang, Xiaolei Liu, Shiqi Xu, Yahui Ding, Yinghui Li, Yingdai Gao, Quan Zhang

**Affiliations:** ^1^ State Key Laboratory of Experimental Hematology, PUMC Department of Stem Cell and Regenerative Medicine, CAMS Key Laboratory of Gene Therapy for Blood Diseases, National Clinical Research Center for Blood Diseases, Haihe Laboratory of Cell Ecosystem, Institute of Hematology and Blood Diseases Hospital, Chinese Academy of Medical Sciences and Peking Union Medical College, Tianjin, China; ^2^ State Key Laboratory of Medicinal Chemical Biology, College of Pharmacy and Tianjin Key Laboratory of Molecular Drug Research, Nankai University, Tianjin, China; ^3^ College of Chemistry, Nankai University, Tianjin, China

**Keywords:** natural product, human hematopoietic stem cell, *ex vivo* expansion, phthalide derivatives, reactive oxygen species (ROS), Levistilide A

## Abstract

Several approaches to expand human hematopoietic stem cells (hHSCs) clinically along with retainable capability of multipotential differentiation have been reported, but only a few have advanced to evaluation in clinical trials, which limits the application of HSC-based therapy. Here we show a phthalide derivative, Levistilide A (LA), can serve as a promising molecule to expand functional human umbilical cord blood (UCB) HSCs *ex vivo*. An in-house screen identified LA out of nine natural products as an outstanding candidate for hHSCs expansion. Additionally, our data indicated that LA treatment not only increased the numbers of phenotype-defined HSCs, but also enhanced their colony formation ability. Xenotransplantation assays showed that LA treatment could maintain unaffected engraftment of hHSCs with multilineage differentiation capacity. Further experiments revealed that LA enhanced the antioxidant activity of hHSCs by reducing intracellular and mitochondrial reactive oxygen species (ROS) levels. The identification of LA provides a new strategy in solving the clinical issue of limited numbers of UCB HSCs.

## Introduction

Development and maintenance of the hematopoietic system rely on a small heterogeneous pool of HSCs which are characterized by the capability of self-renewal and multipotent differentiation (Seita and Weissman, 2010). For individuals with defects in the hematopoietic system, such as hematological malignancies, hematopoietic stem cell transplantation (HSCT) has become the most practical form of stem cell therapy currently (Chivu-Economescu and Rubach, 2017). Hematopoietic stem and progenitor cells (HSPCs) in the graft will generate healthy cells to cure or replace the impaired cells. Superior to bone marrow (BM) and mobilized peripheral blood (mPB), UCB collections can be cryopreserved for over 20 years with efficient recovery of HSPCs after being thawed, and require lower human leukocyte antigen (HLA)-matching (Ballen et al., 2013). However, widespread use of UCB is still limited by two major obstacles: an insufficient dose of HSPCs in a single UCB collection, and subsequent delay of immune recovery post-transplantation (Ballen et al., 2013; Huang et al., 2019). Thus, the top priority at present for UCB transplantation is to develop efficient methods to expand HSPCs *ex vivo* functionally and in turn improve the outcomes of transplantation.

To overcome the aforementioned obstacles, various strategies of expanding HSPCs *ex vivo* have been tested in the laboratory and even in the clinic, including genetic or epigenetic operations, modified culture systems with distinct combinations of cytokines such as stem cell factor (SCF), FMS-like tyrosine kinase-3 ligand (FLT3-L), and thrombopoietin (TPO), and also small molecules targeting certain signaling pathways (Zimran et al., 2021). However, small molecules show more advantages in aspects of safety, operational feasibility, expansion efficiency, and economy (Zhang et al., 2012). As exemplified by the surprising successes demonstrated for StemRegenin 1 (SR1) and UM171, the potential of small molecules to expand HSPCs has gradually been tapped. *Ex vivo* expansion by small molecules can either improve the homing of engrafted HSPCs (Christopherson et al., 2007; Goessling et al., 2011) or expand the absolute number of HSPCs in UCB collections (Fares et al., 2015; Zimran et al., 2021). SR1 (Boitano et al., 2010) and UM171 (Fares et al., 2014) are two potent small molecules that can preferentially expand hematopoietic progenitor cells (HPCs) and long-term HSCs, respectively, *ex vivo*. These approaches will ultimately contribute to improved engraftment of hHSCs.

The functionality of HSCs is affected by intrinsic regulations such as transcription factors and epigenetic modifiers, and extrinsic cues from the BM environment, including ionizing radiation (IR), chemotoxicity, and particularly the surrounding metabolic status (Boulais and Frenette, 2015; Testa et al., 2016). IR- or chemotherapy-induced BM injury leads to defects in HSCs and then senescence, which is caused by increased ROS and oxidative DNA damage (Wang et al., 2010; Wang et al., 2006). Similarly, metabolic status has a great influence on HSCs’ functions by regulating the ROS level of the cell (Testa et al., 2016; Vlaski-Lafarge and Ivanovic, 2015). Different from the environment of downstream progenitors, HSCs are settled in a hypoxic BM environment, in which anaerobic metabolism is the main source of energy (Suda et al., 2011). An elevated intracellular ROS level increases DNA damage, which can promote the expression of cell-cycle inhibitors, causing senescence and loss of functions of HSCs (Yahata et al., 2011). HSCs are thus highly sensitive to oxidative stress (Suda et al., 2011), and their quiescence and functions must be maintained by low oxygen levels both intracellularly and mitochondrially. Therefore, many antioxidants such as ferulic acid, chlorophyllin, and Nicotinamide (NAM) have been developed to protect HSCs from excessive oxidative stress and ultimately to maintain self-renewal and the multipotent differentiation potential of HSCs (Hinge et al., 2010; Zeng-Chun MA, 2010; Baena-Gomez et al., 2015; Suryavanshi et al., 2015; Hwang and Song, 2017), which verifies an important link between ROS and HSCs.

Based on our previous research work (Gao et al., 2015; Xie et al., 2015; Ding et al., 2020; Li et al., 2020; Li et al., 2021), both the platform for chemical compound screening and the following efficacy evaluation of the *ex vivo* expansion of HSCs have been maturely established and put into service to find novel promising candidate compounds. In this study, we screened an in-house collection of natural products to find potential candidates able to expand functional long-term HSCs *ex vivo*. Among these compounds, LA, a dimer of a phthalide derivative, exhibited superior activity in expanding phenotype defined HSPCs and long-term HSCs proportionally and quantitatively. Moreover, LA maintained the engraftment capacity with uncompromised multipotency of hHSCs in NOG mice, suggesting the potential of LA for the *ex vivo* expansion of human UCB stem cells with functionally validated long-term repopulating capability.

## Materials and Methods

### Compounds

All compounds (**1**–**9**) are >95% pure by HPLC analysis.

Compound **1** (neocnidilide): Purity: 98.3% by HPLC. ^1^H NMR (400 MHz, CDCl_3_) δ 6.75 (s, 1H), 4.08­3.88 (m, 1H), 2.48 (s, 1H), 2.40­2.10 (m, 2H), 1.97 (m, 2H), 1.74 (d, J = 5.8 Hz, 2H), 1.58­1.44 (m, 2H), 1.39 (m, 3H), 1.25 – 1.07 (m, 1H), 0.96 – 0.84 (m, 3H). ^13^C NMR (100 MHz, CDCl_3_) δ 170.4, 135.3, 131.3, 85.5, 43.2, 34.5, 27.7, 25.5, 25.1, 22.7, 20.9, 14.0. HRMS(ESI) calculated for C_12_H_18_NaO_2_^+^ [M + Na]^+^: 217.1199, found 217.1205.

Compound **2** (levistilide A, LA): Purity: 98.8% by HPLC. ^1^H NMR (400 MHz, CDCl_3_) δ 7.34 (d, J = 6.6 Hz, 1H), 5.06 (t, J = 7.9 Hz, 1H), 4.99 (t, J = 7.5 Hz, 1H), 3.24 (d, J = 8.9 Hz, 1H), 2.98 (dd, J = 6.5, 2.1 Hz, 1H), 2.54 (t, J = 7.7 Hz, 1H), 2.28 (q, J = 7.6 Hz, 2H), 2.24 – 2.14 (m, 3H), 2.13 – 1.98 (m, 2H), 1.97 – 1.82 (m, 2H), 1.63 – 1.40 (m, 6H), 1.30 (ddd, J = 12.2, 4.7, 2.6 Hz, 1H), 0.98 – 0.87 (m, 6H). ^13^C NMR (100 MHz, CDCl_3_) δ 168.6, 165.0, 155.1, 150.6, 148.1, 142.2, 134.3, 126.7, 112.3, 108.7, 47.7, 41.7, 41.6, 38.4, 31.2, 29.1, 28.1, 27.6, 25.9, 22.4, 22.4, 19.9, 14.1, 13.9. HRMS (ESI) calculated for C_24_H_29_O_4_^+^ [M^+^H]^+^: 381.2060, found 381.2062.

Compound **3**: ^1^H NMR (400 MHz, CDCl3) δ 5.32 – 5.22 (m, 1H), 4.48 (dt, J = 6.2, 1.7 Hz, 1H), 3.95 (ddd, J = 9.4, 6.0, 3.1 Hz, 1H), 3.43 (s, 2H), 2.64 – 2.44 (m, 2H), 2.34 (q, J = 7.6 Hz, 2H), 2.09 (dtd, J = 13.7, 5.3, 3.1 Hz, 1H), 1.88 (dddd, J = 13.7, 9.6, 8.3, 6.1 Hz, 1H), 1.49 (h, J = 7.4 Hz, 2H), 0.94 (t, J = 7.4 Hz, 3H); ^13^C NMR (100 MHz, CDCl_3_) δ 169.12, 152.86, 148.10, 125.98, 114.53, 71.92, 68.09, 28.21, 26.77, 22.39, 19.32, 13.91.

Compound **4**: ^1^H NMR (400 MHz, CDCl3) δ 6.39 – 6.09 (m, 1H), 5.91 (dq, J = 9.5, 3.2 Hz, 1H), 4.92 (dd, J = 7.7, 3.9 Hz, 1H), 2.63 – 2.41 (m, 4H), 1.88 (dtt, J = 14.3, 6.9, 3.0 Hz, 1H), 1.59­1.48 (m, 1H), 1.39 (tdd, J = 17.7, 8.5, 4.6 Hz, 4H), 0.91 (td, J = 7.1, 2.6 Hz, 3H); ^13^C NMR (100 MHz, CDCl_3_) δ 171.39, 161.53, 128.45, 124.66, 117.04, 82.64, 32.05, 26.87, 22.58, 22.43, 20.94, 14.00.

Compound **5**: ^1^H NMR (400 MHz, CDCl3) δ 7.85 (dq, J = 7.7, 1.0 Hz, 1H), 7.68 – 7.60 (m, 2H), 7.48 (ddt, J = 7.5, 6.4, 1.1 Hz, 1H), 5.62 (t, J = 7.8 Hz, 1H), 2.47­2.38 (m, 2H), 1.53 (h, J = 7.4 Hz, 2H), 0.96 (td, J = 7.4, 0.8 Hz, 3H); ^13^C NMR (100 MHz, CDCl_3_) δ 167.29, 145.80, 139.63, 134.31, 129.40, 125.25, 124.47, 119.72, 109.55, 27.85, 22.58, 13.87.

Compound **6**: ^1^H NMR (400 MHz, CDCl3) δ 5.30 (t, J = 7.9 Hz, 1H), 4.61 (dd, J = 4.2, 2.3 Hz, 1H), 4.05 (ddt, J = 8.0, 4.0, 2.1 Hz, 1H), 3.40 (d, J = 2.8 Hz, 1H), 2.83 (d, J = 4.1 Hz, 1H), 2.65 (dddd, J = 18.4, 7.3, 5.6, 1.7 Hz, 1H), 2.45 – 2.28 (m, 3H), 2.13 (ddt, J = 13.7, 7.9, 5.8 Hz, 1H), 1.82 (tdd, J = 9.9, 6.4, 2.4 Hz, 1H), 1.49 (h, J = 7.3 Hz, 2H), 0.94 (t, J = 7.4 Hz, 3H); ^13^C NMR (100 MHz, CDCl_3_) δ 169.43, 153.38, 148.32, 125.47, 114.59, 67.41, 63.56, 28.21, 25.76, 22.39, 18.48, 13.91.

Compound **7**: ^1^H NMR (400 MHz, CDCl3) δ 7.85 (d, J = 7.6 Hz, 1H), 7.64 (td, J = 7.5, 1.1 Hz, 1H), 7.49 (t, J = 7.5 Hz, 1H), 7.42 (dd, J = 7.6, 0.9 Hz, 1H), 5.45 (dd, J = 7.9, 4.1 Hz, 1H), 2.02 (dddd, J = 14.2, 10.0, 5.8, 4.1 Hz, 1H), 1.73 (dddd, J = 14.5, 10.0, 7.9, 4.7 Hz, 1H), 1.52­1.25 (m, 4H), 0.87 (t, J = 7.1 Hz, 3H); ^13^C NMR (100 MHz, CDCl_3_) δ 170.71, 150.15, 133.99, 129.03, 126.12, 125.63, 121.80, 81.47, 34.44, 26.89, 22.44, 13.87.

Compound **8**: ^1^H NMR (400 MHz, CDCl3) δ 6.24 (dt, J = 9.6, 2.1 Hz, 1H), 5.97 (dt, J = 9.7, 4.2 Hz, 1H), 5.20 (t, J = 8.0 Hz, 1H), 2.57 (td, J = 9.5, 1.7 Hz, 2H), 2.49 – 2.39 (m, 2H), 2.34 (q, J = 7.6 Hz, 2H), 1.47 (h, J = 7.3 Hz, 2H), 0.92 (t, J = 7.4 Hz, 3H); ^13^C NMR (100 MHz, CDCl_3_) δ 167.70, 148.62, 147.19, 130.01, 124.02, 117.12, 113.02, 28.19, 22.47, 18.58, 13.85.

Compound **9**: ^1^H NMR (400 MHz, CDCl3) δ 7.51 (d, J = 6.7 Hz, 1H), 6.16 (dt, J = 9.6, 2.0 Hz, 1H), 5.92 (dt, J = 9.6, 4.2 Hz, 1H), 4.62 (dd, J = 8.7, 6.8 Hz, 1H), 3.10 (dt, J = 7.0, 2.4 Hz, 2H), 2.52­2.45 (m, 1H), 2.33 – 2.24 (m, 2H), 2.23­1.91 (m, 6H), 1.70 – 1.63 (m, 1H), 1.51­1.41 (m, 1H), 1.39 – 1.20 (m, 4H), 1.17 – 1.07 (m, 1H), 0.88 (t, J = 7.1 Hz, 3H), 0.81 (t, J = 7.4 Hz, 3H); ^13^C NMR (100 MHz, CDCl_3_) δ 170.57, 164.37, 161.88, 148.58, 145.02, 133.09, 129.48, 125.65, 116.70, 107.64, 89.76, 50.18, 42.85, 36.75, 28.46, 28.26, 27.13, 22.73, 21.29, 21.19, 17.06, 14.27, 13.67.

### Animals

Female NOD/Shi-scid/IL2Rγnull (NOG) mice (6-7 weeks) were purchased from Charles River and were housed in specific-pathogen-free (SPF) conditions, with free access to food and water. All animal protocols were approved by the Animal Care and Use Committee of State Key Laboratory of Experimental Hematology, Institute of Hematology and Blood Diseases Hospital.

### Human UCB CD34^+^ Cells Collection and Processing

Samples were collected from consenting donors by Shandong Qilu Stem Cell Engineering Co., Ltd. in accordance with the declaration of Helsinki and approved by the Ethics Review Board of the Institute of Hematology and Blood Diseases Hospital, Chinese Academy of Medical Sciences. Fresh collected human umbilical cord blood CD34^+^ cells were isolated using LS Column and QuadroMACS Separator (Miltenyi Biotec), according to the manufacturer’s protocol after collecting CD34 MicroBead-labeled cells by magnetic activated cells sorting (MACS) CD34 MicroBeads (Miltenyi Biotec).

### *Ex vivo* HSC Expansion

For compound screening assays and mechanism research, fresh human UCB CD34^+^ cells were cultured in HSC expansion medium consisting of Iscove’s Modified Dulbecco’s Medium (IMDM, Gibco) supplemented with 10% fetal bovine serum (FBS, Gibco), 100 ng/ml human stem cell factor (hSCF, PeproTech), 100 ng/ml human Fms-related tyrosine kinase three ligand (hFlt3-L, PeproTech), 100 ng/ml human thrombopoietin (hTPO, PeproTech), and 1% penicillin-streptomycin (P/S, Sigma-Aldrich). Isolated human CD34^+^ UCB cells were resuspended in HSC expansion medium (5.3 × 10^4^ cells/mL) before being seeded into 96 well plates (Corning). Small molecule compounds were dissolved in dimethyl sulfoxide (DMSO, Sigma-Aldrich) and stored as stock solutions. Stock solutions were diluted to working solutions at the desired concentration by HSC expansion media, and then added into cell suspension in 96 well plates. Each well contained 190 μL cell suspension (1 × 10^4^ cells) and 10 μ L small molecule compound working solution, which were fully blended. The final concentration of DMSO did not exceed 0.1% (v/v). Cells were incubated at 37°C with 5% CO_2_ for 7 days. For further phenotype and function assays, fresh human UCB CD34^+^ cells were cultured in HSC expansion medium consisting of StemSpan Serum-free Expansion Medium (SFEM, StemCell Technologies), 100 ng/ml hSCF, 100 ng/ml hFlt3-L, 100 ng/ml hTPO, 1% P/S, and supplemented with vehicle control (0.05% DMSO, v/v) or SR1 (Selleck) [1 µM] or LA [10 µM] or a combination of LA [10 µM] + SR1 [1 µM]. Isolated human CD34^+^ UCB cells were resuspended in HSC expansion medium (2 × 10^6^ cells/mL) before being seeded into 24 well plates (Corning). Each well contained 100 μL cell suspension (2 × 10^5^ cells), 100 μ L small molecule compound working solution, and 1800 µL expansion medium, which were fully blended. For transplantation experiments, human CD34^+^CD38^−^CD45RA^−^CD90^+^ cells were sorted into 96 well plates at 300 cells per well in 250 µL culture system. HSC expansion media composed of SFEM, 100 ng/ml hSCF, 100 ng/ml hFlt3-L, 100 ng/ml hTPO, 1% P/S, and supplemented with vehicle control (0.05% DMSO, v/v) or SR1 [1 µM] or LA [10 µM] or a combination of LA [10 µM] + SR1 [1 µM]. Cells were incubated at 37°C with 5% CO_2_ for 4 days.

### Flow Cytometry Analysis

UCB CD34^+^ cells were seeded at 1 × 10^4^ cells per well in the presence of chemical compounds. Total expanded cells were collected, and the live cells were counted using trypan blue and an automated cell counter (Bio-Rad, TC20) after 4–7 days of culture. Cell phenotypes in expanded cells were stained at 4°C for 30–60 min in PBS supplemented with a combination of the following antibodies and fluorophores: APC-labeled anti-human CD34 (BD; 555824), PE-Cy7-labeled anti-human CD38 (BD; 560,677), APC-H7-labeled anti-human CD45RA (BD; 560,674), PerCP-Cy5.5-labeled anti-human CD90 (BD; 561557), and PE-labeled anti-human CD49f (BD; 555736). Following a wash step, stained cells were analyzed using an LSRII (BD) or FACS CantoII (BD) flow cytometer. The absolute numbers of input cells were calculated based on FACS data of freshly isolated CD34^+^ cells and the initial seeding number in the culture. The absolute numbers of output cells were obtained by multiplying the viable cell counts of expanded progeny by FACS proportion data.

### Colony Forming Cell Assay (CFC)

The concentration of cultured human UCB CD34^+^ cells was adjusted to 50 µL/1000 initial cells in Iscove’s Modified Dulbecco’s Medium (IMDM). Frequencies of colony-forming cells were estimated by plating 10 μL cell suspension (equivalent to 200 initial cells) in 1 ml MethoCult™ GF H4434 (StemCell Technologies) in 6 well plates (Corning). After 14 days in culture, plates were visually scored for CFU-GM (colony-forming unit-granulocyte/macrophage), CFU-E (colony-forming unit-erythrocyte), BFU-E (burst-forming unit-erythroid), and CFU-GEMM (colony-forming unit granulocyte/erythrocyte/macrophage/megakaryocyte).

### Cobblestone Area Forming Cell Assay (CAFC)

The frequency of Week 5 CAFC was determined using a limiting dilution assay. Cultured UCB CD34^+^ cells were seeded on cryopreserved irradiated (8000 cGy) M2-10B4 bone marrow stromal cells (ATCC) in flat-bottomed collagen-coated 96 well plates at five different concentrations (63, 125, 250, 500, and 1000 cells) with 12 replicates per dilution. For assessment of CAFC after 5 weeks of culture, all wells were scored microscopically. Wells were scored as being positive for the presence of at least one cobblestone area (CA, tightly knit group of phase-dark, angular cells in the stroma). The CA-forming cell frequencies were calculated by ELDA software (http://bioinf.wehi.edu.au/software/elda/).

### Transplantation and Monitoring of Human HSCs in NOG Mice

At 7–8 weeks of age, mice were irradiated at a dose of 250 cGy 4 h prior to transplantation. Experiments were conducted in sodium pentobarbital-anesthetized mice. For the uncultured group, freshly sorted UCB DAPI^−^CD34^+^CD38^−^CD45RA^−^CD90^+^ cells were counted and resuspended in 300 cells/25 µL PBS per mouse and injected into mouse tibiae. For the compound-treated groups, 3000 sorted DAPI^−^CD34^+^CD38^−^CD45RA^−^CD90^+^ cells were cultured for 4 days in the presence of chemical compounds as previously described. The expanded bulk-cell cultures were washed by PBS and adjusted to 300 equivalent initial cells/25 µL PBS per mouse and injected into mouse tibiae. Human cell chimerism was calculated at 4-, 8-, and 12-weeks post transplantation in the peripheral blood and 16-weeks post transplantation in bone marrow, using FITC-labeled anti-human CD45 (BD; 555482). At least 2% donor chimerism in PB or 15% in BM was determined as the threshold for positive engraftment. Multilineage reconstitution in BM was analyzed 16 weeks post-transplantation, using APC-labeled anti-human CD33 (BD; 551378), PE-Cy7-labeled anti-human CD3 (BD; 557851), PerCP-Cy5.5-labeled anti-human CD56 (BD; 560842), APC-labeled anti-human CD235a (BD; 561,775), and PE-labeled anti-human CD41a (BD; 557297).

### ROS Assay

The cultured UCB CD34^+^ cells treated with LA, SR1, DMSO, or LA plus SR1 were resuspended at a density of 5 × 10^5^ to 1 × 10^6^ cells/mL. Prior to adding working solution to cell suspensions prepared according to the manufacturer’s instructions (Fluorometric Intracellular ROS Kit, Siga-Aldrich, cat: MAK144; MitoSOX™ Red mitochondrial superoxide indicator, Invitrogen, cat: M36008), the extracellular fluorescein labeled antibodies were first labeled for 30 min at 4°C. The fluorescence intensity of ROS was measured within 2 h by FACSArialll (BD) flow cytometer. The median level of MFI (mean fluorescence intensity) of ROS was obtained after Downsample in software FlowJo 10 (BD) of each sample to the same measuring cell number (for intracellular ROS: downsample CD34^+^ subpopulation to 424,800 cells and CD34^+^CD38^−^ subpopulation to 47,808 cells; for MitoSox: downsample CD34^+^ subpopulation to 111,430 cells and CD34^+^CD38^−^ subpopulation to 11,780 cells).

### RNA Extraction

Cultured human UCB CD34^+^ cells were lysed, and RNA was extracted using Trizol. RNA degradation and contamination were monitored on 1% agarose gels. RNA purity was checked using the NanoPhotometer spectrophotometer (IMPLEN, CAUnited State). RNA concentration was measured using Qubit RNA Assay Kit with Qubit 2.0 Flurometer (Life Technologies, CA, United States). RNA integrity was assessed using the RNA Nano 6000 Assay Kit of the Bioanalyzer 2100 system (Agilent Technologies, CA, United States).

### RNA-Sequencing

A total amount of 3 μg RNA per sample was used as input material for the RNA sample preparations. Sequencing libraries were generated using NEBNext UltraTM RNA Library Prep Kit for Illumina (NEB, United States) following manufacturer’s recommendations, and index codes were added to attribute sequences to each sample. The PCR products were purified with AMPure XP system (Beckman Coulter, Beverly, MA, United States) and library quality was assessed on the Agilent Bioanalyzer 2100 system. The clustering of the index-coded samples was performed on a cBot Cluster Generation System using TruSeq PE Cluster Kit v3-cBot-HS (Illumina) according to the manufacturer’s instructions. After cluster generation, the library preparations were sequenced on an Illumina Hiseq platform and 125 bp/150 bp paired-end reads were generated. After mapping to the reference genome and quantification, DESeq2 (R package, 1.10.1) was used to analyze the differentially expressed genes. |Log2(FoldChange)| > 0.0 and the adjusted *p*-value (qadj) < 0.05 were used as a standard of cutting off for significance. The significantly changed genes were used in GO. GO terms results of molecules function (MF) were summarized and clustered based on semantic similarity measures using the online tool REVIGO (Supek et al., 2011). Total gene list was used in GSEA analysis, and gene sets used were searched from the GSEA gene set database (http://www.gsea-msigdb.org/gsea/msigdb/index.jsp).

### Quantitative Real-Time PCR (qRT-PCR)

Reverse transcription of extracted RNA was performed using M-MLV Reverse transcriptase (Invitrogen, 28025-013) to obtain cDNA, following the manufacturer’s instructions. qRT-PCR was then performed in a 384 well plate PCR reaction system using TB Green Premix Ex Taq (Tli RNaseH lusP) (Takara Bio, RR420A), according to the manufacturer’s instructions. The PCR was run in QuantStudio six Flex (Thermo Fisher Scientific) selecting standard procedure for two-step PCR amplification: pre-denaturation (one cycle of 95°C for 30 s) and PCR reaction (40 cycles of 95°C for 5 s and then 60°C for 30 s). The expression of target transcripts was normalized to that of internal control GAPDH, and their relative expressions were processed based on their 2^−ΔΔCt^. The primers for target genes are listed in Supporting Information 1.

### Statistical Analysis

All data are presented as the mean ± standard deviation (SD) and all statistical analyses were done using the software Graphpad Prism version 8.4.0 (GraphPad Software). Two tailed Student’s t-test was used to generate *p* values for most of the data sets and *p*-values < .05 were considered statistically significant. Additionally, *p < .05, **p < .01, ***p < .001, ****p < .0001, ns = no significance. Chi-square test was used and performed by ELDA software in CAFC assay.

## Results

### Screening of Nine Phthalide Derivatives That Can Expand HSPCs From UCB CD34^+^Cells

**FIGURE 1:**
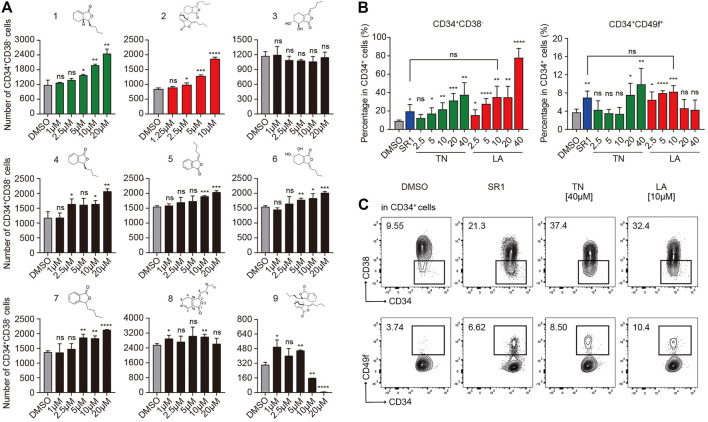
Identification of proliferative activities of phthalide derivatives 1 to 9 and the optimal working concentration of candidate compounds for *in vitro* expansion of human UCB CD34^+^ cells. **(A)** The structures of phthalide derivatives 1 to 9 and the corresponding absolute number of CD34^+^CD38^−^ cells after a serum-based 7-d culture in gradient concentrations as specified (*n* = 6). **(B)** The percentages of CD34^+^CD38^−^ and CD34^+^CD49f^+^ subpopulations in CD34^+^ cells after a serum-based 7-d culture supplemented with DMSO (0.05%, v/v), SR1 (1 µM), Levistilide A (LA, 2.5 –40 µM), or Tetrahydrophthalide Neocnidilide (TN, 2.5 –40 µM) (*n* = 5). **(C)** Representative FACS profiles of CD34^+^CD38^−^ and CD34^+^CD49f^+^ subpopulations in cultured CD34^+^ cells as described in **(B)**. All data represent the means ± SD. Compared with fresh unless specified. *p < 0.05, **p < 0.01, ***p < 0.001, ****p < 0.0001 and ns = not significant by two-tailed unpaired Students’ *t*-test.

To confirm the activity of expanding HSPCs among candidate compounds, primary human CD34^+^ cells isolated from UCB using MACS were seeded into 96 well plates (1×10^4^ cells per well) in medium (IMDM +10%FBS +100 ng/ml hSCF +100 ng/ml hTPO +100 ng/ml hFLT3-L + 1%P/S) supplemented with each compound (1–20 µM) or DMSO (0.05%, v/v, used in subsequent experiments unless otherwise noted), respectively. After 7 days incubation, the mixture of expanded cells was processed and the absolute number of HSPCs (Figure 1A) were analyzed by flow cytometry based on immunophenotype: CD34^+^CD38^−^. As is shown in Figure 1A, compound **2** (Levistilide A, LA) exhibited the most potent activity to expand HSPCs, followed by compound **1** (Tetrahydrophthalide Neocnidilide, TN), and next followed by other compounds **4**–**7**. LA is the dimer of compound **8** ((Z)-ligustilide, a common phthalide), but the monomer **8** demonstrated nearly no expansion activity. It appears that the dimer is more efficient than the monomer on expanding HSPCs and monomer **4** to **7** might be potential small molecular fragments to form effective compounds, similarly to monomer **8**. Significantly, the dimer **9** nearly lost potency to expand HSPCs. It is noteworthy that dimer **2** illustrates that the 6,7-alkene unit undergoes 4 + 2 cycloaddition as the dienophile with the monomer **8** 1,3-diene system, while dimer **9** is derived from 4 + 2 cycloaddition of the cross-conjugated 3,8-alkene. Therefore, for dimers, the pattern of 4 + 2 cycloaddition could be important for their HSPC expansion activity. Based on the results above, TN and LA were identified as candidate compounds for HSPC expansion deserving further functional investigation.

### Identification of Optimal HSPC Expansion Concentration of LA

To investigate the optimal expansion concentration of candidate TN and LA, UCB CD34^+^ cells were incubated for 7 days in medium (IMDM +10%FBS +100 ng/ml hSCF +100 ng/ml hTPO +100 ng/ml hFLT3-L + 1%P/S) with TN or LA (2.5, 5, 10, 20, or 40 μM, respectively). SR1 (1 μM, used in subsequent experiments unless otherwise noted) was used as a positive control and equivalent DMSO as a negative control. Flow cytometry analysis was performed to analyze the percentage of subpopulations in live cells based on the expression of cell surface markers: CD34, CD38, and CD49f (Notta et al., 2011), which were always used to define a more primitive subpopulation of HSCs. Both LA and TN led to a dose-dependent increase in the percentage of CD34^+^CD38^−^ HSPCs (Figure 1B, left panel). As for CD34^+^CD49f^+^ HSCs, LA expanded cells in the concentration range of 2.5–10 μM, while TN worked at a concentration of at least 20 µM (Figure 1B, right panel). This indicated that LA was more potent expanding more primitive cell subsets than TN. SR1 treatment led to a twofold increase compared to DMSO in the percentage of both CD34^+^CD38^−^ HSPCs and CD34^+^CD49f^+^ HSCs (20 vs. 10% and 8 vs. 4%, respectively), which was similar to the expansion effects of LA at 10 µM (Figures 1B,C). Thus, our group identified LA as the most potent out of nine phthalide derivatives, and 10 µM (used in subsequent experiments) as the optimal concentration to expand HSPCs initiated with UCB-CD34^+^ cells.

### LA Enhanced Expansion of Phenotype-Defined Long-Term HSCs *in vitro*

**FIGURE 2:**
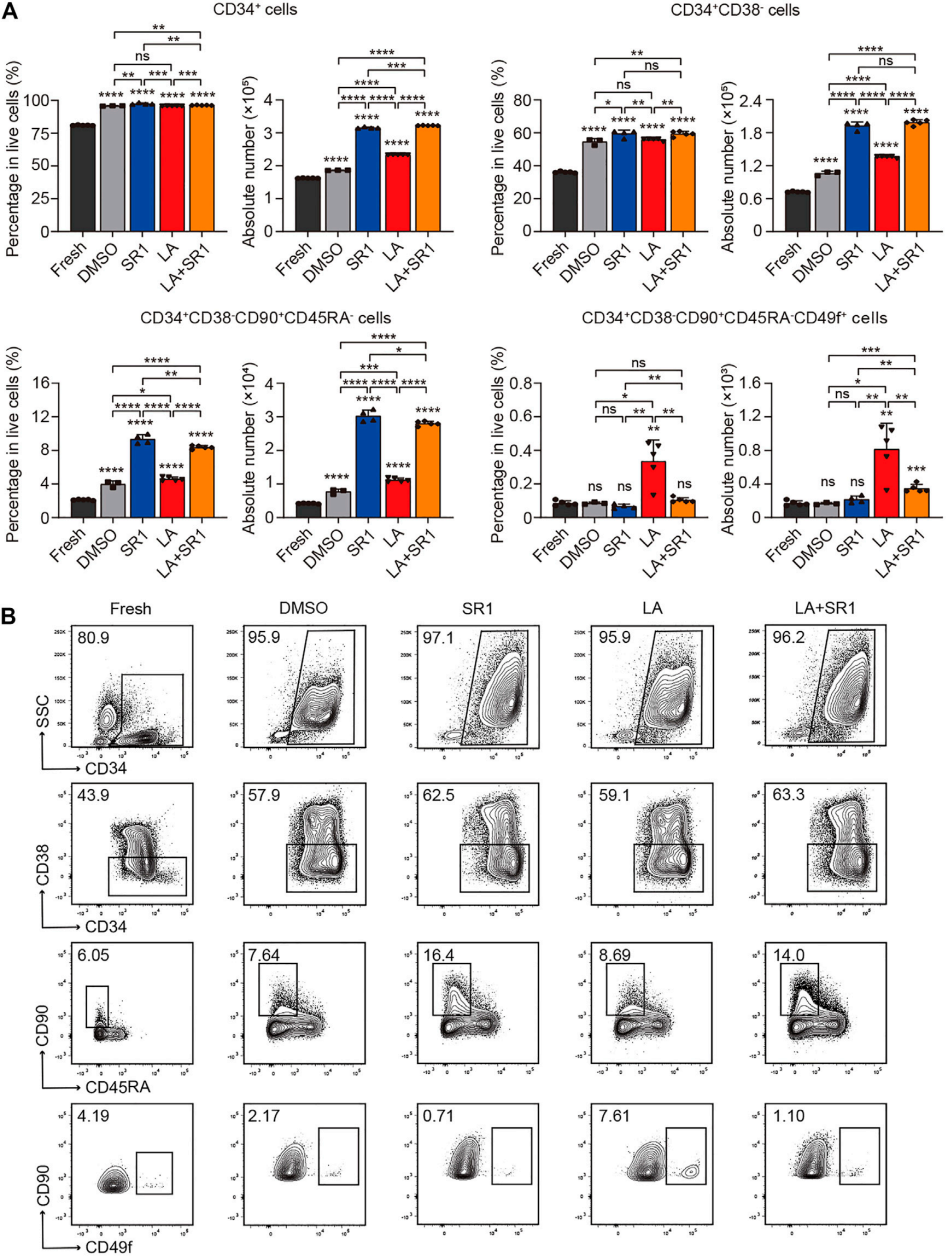
LA promoted expansion of UCB HSCs *in vitro*. **(A)** The percentages in live cells and absolute number of CD34^+^, CD34^+^CD38^−^, CD34^+^CD38^−^CD45RA^−^CD90^+^ and CD34^+^CD38^−^CD45RA^−^CD90^+^CD49f^+^ subpopulations before (fresh, *n* = 5) and after a serum-free 4 days culture with DMSO (0.05%, v/v) (*n* = 3), SR1 (1 µM) (*n* = 4), LA (10 µM) (*n* = 5), or LA + SR1 (*n* = 5). All data represent the means ± SD. Compared with fresh unless specified. *p < 0.05, **p < 0.01, ***p < 0.001, ****p < 0.0001 and ns = not significant by two-tailed unpaired Students’ *t*-test. **(B)** Representative FACS profiles of phenotypically defined subpopulations in cultured CD34 ^+^ cells as described in **(A)**.

It is known that CD34, CD38, CD90, CD45RA, and CD49f are common cell surface markers used for identifying human HSPCs *in vitro* and *in vivo*, and the detection of different combinations of these markers is a close estimate of HSCs (Tajer et al., 2019). Thus, we compared the difference of percentage in live cells and absolute number between the fresh UCB CD34^+^ cells and cultured cells (treated with DMSO, SR1, LA or LA + SR1, respectively) by flow cytometry analysis to determine the expansion activity of LA alone and LA + SR1 at 10 µM on certain subpopulations of HSCs. When in a serum-free culture system (SFEM+100 ng/ml hSCF+100 ng/ml hTPO+100 ng/ml hFLT3-L+1%P/S) for 4 days, LA treatment significantly increased the number of CD34^+^ cells and CD34^+^CD38^−^ cells rather than in percentage compared with fresh or DMSO, while it increased primitive CD34^+^CD38^−^CD45RA^−^CD90^+^ HSCs and CD34^+^CD38^−^CD45RA^−^CD90^+^CD49f^+^ long-term HSCs obviously in both percentage and cell number, especially the latter subset (Figure 2A). LA incubation increased CD34^+^CD38^−^CD45RA^−^CD90^+^CD49f^+^ cells up to sixfold compared with the fresh or DMSO, which is significantly more effective than SR1 treatment (Figure 2A). Notably, combining LA with SR1 could significantly improve the problem of insufficient SR1 expansion in counts of CD34^+^CD38^−^CD45RA^−^CD90^+^CD49f^+^ cells (Figure 2A). When the culture time was prolonged to 7 days in this system, LA treatment could significantly expand both the percentage and cell number of these 4 cell populations, the fold change of which was nearly similar with that of 4-day culture. Additionally, 7-day LA treatment led to a 10-fold increase in the cell number of CD34^+^ cells, CD34^+^CD38^−^ cells, or CD34^+^CD38^−^CD45RA^−^CD90^+^ cells compared with 4-day culture, while up to 25-fold in cell number for CD34^+^CD38^−^CD45RA^−^CD90^+^CD49f^+^ cells and up to twofold in percentage (Figure 2A, Supplementary Figure S1A). Additionally, the expansion effect of SR1 on CD34^+^CD38^−^CD45RA^−^CD90^+^CD49f^+^ cells was also manifested by prolonging the culture time (Figure 2A, Supplementary Figure S1A). When it comes to a 7-d serum-based culture, we found that treatment with DMSO, SR1, LA, LA + SR1 is inferior to that of serum-free culture neither in percentage nor in cell number (Figure 2A, Supplementary Figure S2A). However, regardless of the culture system with or without serum, the expansion of CD34^+^CD38^−^CD45RA^−^CD90^+^CD49f^+^ long-term HSCs by LA was significantly superior to fresh or DMSO in both the percentage and cell number (Figure 2A, Supplementary Figure S2A). Representative FACS profiles of phenotypically defined subpopulations can be seen in Figure 2B, Supplementary Figure S1B, and Supplementary Figure S2B. These results indicated that incubation with LA can significantly expand phenotype defined HSPCs, in particular long-term HSCs.

### LA Enhanced Colony Formation Capacity of HSCs

**FIGURE 3:**
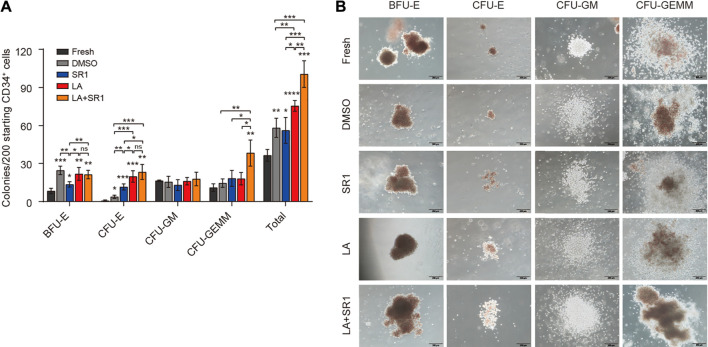
LA enhanced the short-term clonogenic capacity of HSPCs *in vitro*. **(A)** Colonies derived from LA-treated (10 µM) cells in serum-free 4-d system following an additional 14-d culture in H4434 methylcellulose (*n* = 3). All data represent the means ± SD. Compared with fresh unless specified. *p < 0.05, **p < 0.01, ***p < 0.001, ****p < 0.0001 and ns = not significant by two-tailed unpaired Students’ *t*-test. **(B)** Representative morphological images of CFU colonies (BFU-E, CFU-E, CFU-GM, CFU-GEMM). Scale bar, 200 µm.

After identifying the *ex-vivo* expansion effect of LA on phenotype-defined HSPCs, we next evaluated the short-term differentiation potential of LA-treated cells in colony-forming cell (CFC) assays. First, we examined the clonogenic capacity of hUCB CD34^+^ cells cultured *in vitro* in serum-free system added with DMSO, SR1, LA, or LA + SR1 for 4 d. It shows that LA-treated cells generated twofold, onefold, and onefold more total colonies than fresh, DMSO, and SR1, respectively. A combination of LA and SR1 can generate more total colonies than LA alone (Figure 3A). The number of erythrocyte colonies by the treatment of both LA and LA + SR1 were significantly higher than that of fresh and SR1. For BFU-E, both LA and LA + SR1 treatment generated significantly increased colonies as compared with fresh and SR1 and there was no significance between LA and LA + SR1. LA could also significantly increase the numbers of total colonies and CFU-E compared with DMSO in the 7-day serum-based culture system (Supplementary Figure S3A). Representative morphological images of different types of colonies are shown in Figure 3B and Supplementary Figure S3B. Then, cobblestone area-forming cell (CAFC) assays were carried out to assess the long-term hematopoietic activity of LA-treated cells initiated with UCB-CD34^+^ cells. Limiting dilution analysis (LDA) showed that 0.88% (1 out of 113) of LA-treated CD34^+^ cells had long-term repopulating activity, which was 1.7 times that of the DMSO group (0.52%, 1 out of 192) (Supplementary Figure S3C), suggesting that LA maintained the long-term viability of HSPCs. Taken together, these results indicated that LA sustains the multipotentiality and long-term repopulating activity of HSPCs and promotes erythroid differentiation during *in vitro* culture.

### LA Maintained Human HSCs Engraftment in a Xenograft Model

Although CFU and CAFC assays are usually used for assessing activities of HSCs and progenitors *in vitro*, xenotransplantation into immunodeficient mice is reported to be a gold standard assay to assess whether cultured cells *in vitro* can maintain the ability of HSCs to indefinitely repopulate all blood cell lineages (Goyama et al., 2015). We thus sorted CD34^+^CD38^−^CD45RA^−^CD90^+^ cells from the fresh and cultured groups (treated with DMSO, SR1, LA, or SR1 plus LA, respectively) initiated with UCB CD34^+^ cells, which were injected respectively into sub-lethally irradiated severe combined immunodeficient NOD/Shi-scid/IL2Rγnull (NOG) mice *via* tail vein.

**FIGURE 4:**
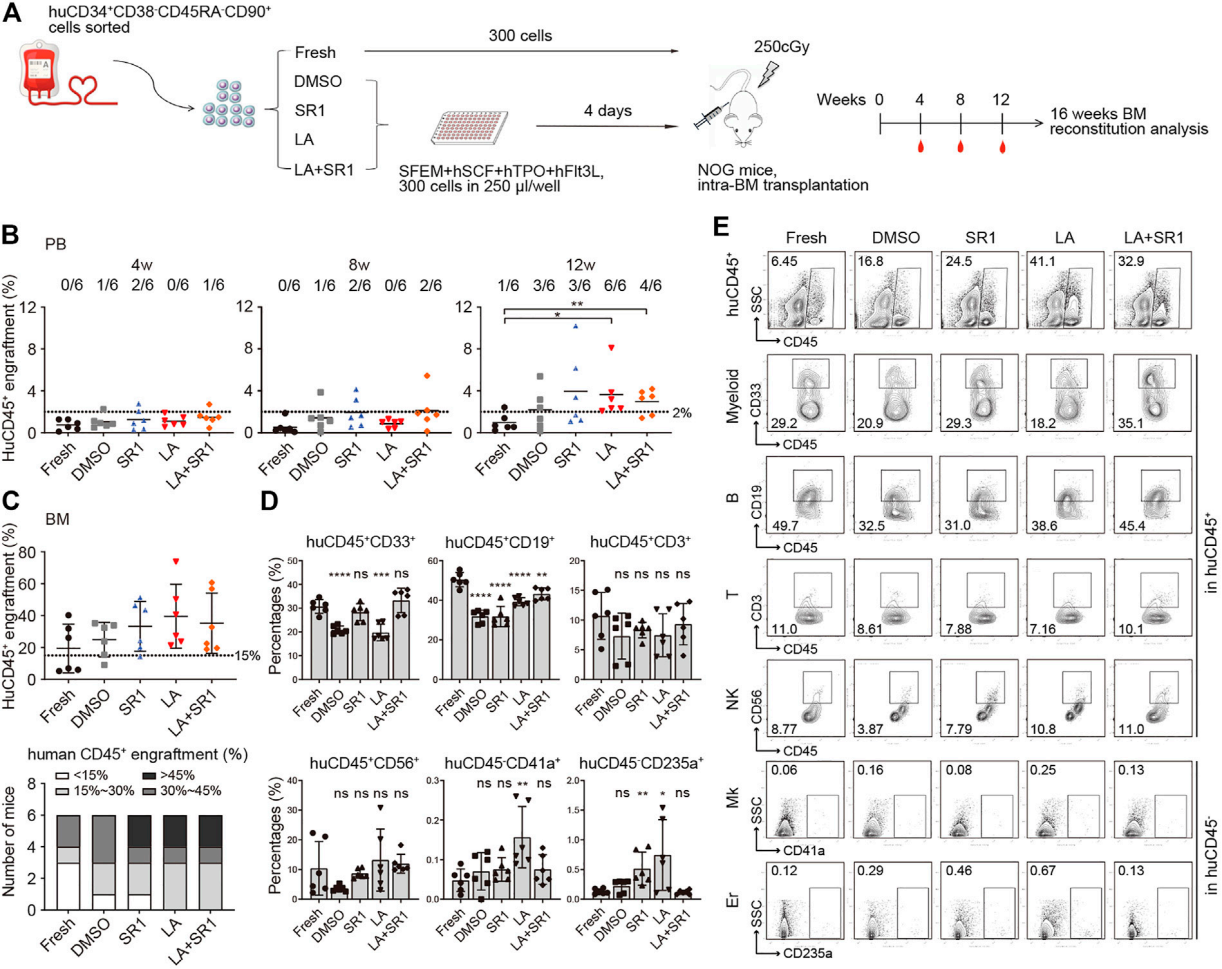
Engraftment and reconstruction of human UCB CD34^+^CD38^−^CD45RA^−^CD90^+^ cells before (fresh) and after a 4-d culture with DMSO, SR1, LA alone, or in combination with SR1 (LA + SR1) in sublethal irradiated NOG mice. **(A)** Schematic representation of xenograft experiment. NOG mice were transplanted with 300 fresh or cultured cells (*n* = 6 mice per group). **(B)** Human engraftment in peripheral blood (PB) of NOG mice was detected at 4-, 8-, and 12-weeks post-transplantation and mice were sacrificed at 16-weeks post-transplantation for bone marrow (BM) analysis. **(B)** The percentage of human CD45^+^ cells in PB of NOG mice. Numbers of mice with ≥2% engraftment percentage are specified. **(C)** The percentage of human CD45^+^ cells in BM of NOG mice (upper panel) and the number of mice at different engraftment levels (<15%; 15%–30%; 30%–45%; >45%) (lower panel) in each group at 16-weeks post-transplantation. **(D)** Levels of myeloid (CD45^+^CD33^+^), B lymphocytes (CD45^+^CD19^+^), T lymphocytes (CD45^+^CD3^+^), natural killer cells (CD45^+^CD56^+^), megakaryocytes (CD45^−^CD41a^+^), and erythroid (CD45^−^CD235a^+^) reconstitution in BM of NOG mice at 16-weeks post-transplantation. **(E)** Representative FACS profiles showing engraftment of human CD45^+^ cells and multilineage reconstruction described in **(C,D)**.

Flow cytometry was performed to measure human cell engraftment in peripheral blood (PB) at 4, 8, and 12 weeks and in BM at 16-weeks post-transplantation. We regarded the level of CD45^+^ human cells not less than 2% in PB and not less than 15% in BM of mice recipients as a baseline of successful engraftment. Although human cells engraftment in PB of recipient NOG showed no significant difference among all groups at both 4- and 8-weeks post-transplantation, some mice in the SR1 group and SR1 plus LA group generated a slightly higher level of engraftment at week 8 (Figure 4B). At 12 weeks, we observed a significant increase of engraftment in the LA group compared to the fresh group. In addition, the SR1 plus LA group also increased the engraftment levels notably when compared with the fresh group, indicating that LA treatment may maintain engraftment of human cells (Figure 4B). These results indicated that LA treatment could maintain normal engraftment of human cells in recipient PB. Consistent with the results analyzed in PB at 12 weeks, when LA was administered, either the LA group or the SR1 plus LA group exhibited a generally higher level of human CD45^+^ cells in BM at 16-weeks post-transplantation (Figure 4C, upper panel), which suggested that LA treatment retained the long-term engraftment of human cells. Except for the LA group and the SR1 plus LA group, there were recipients that did not achieve positive engraftment (the percentage of human CD45^+^ cells less than 15%) in the fresh group (3 out of 6), the DMSO group (2 out of 6), or the SR1 group (1 out of 6) (Figure 4C, lower panel), which implied an unaffected engraftment of human cells after culturing with LA *ex vivo*.

Assessment of hematopoietic reconstitution in BM at 16-weeks post-transplantation was subsequently conducted, including total leukocytes (CD45^+^), myeloid cells (CD45^+^CD33^+^), B lymphoid cells (CD45^+^CD19^+^), T lymphoid cells (CD45^+^CD3^+^), natural killer cells (NK cells, CD45^+^CD56^+^), megakaryocytes (CD45^−^CD41a^+^), and erythroid cells (CD45^−^CD235a^+^). As shown in Figures 4D,E, multiple lineages could be observed in both the LA group and the SR1 plus LA group, suggesting that LA treatment did not affect the multilineage differentiation potential of HSPCs. Differentiation into megakaryocytes and erythroid cells was significantly improved by LA treatment (Figure 4D), which was consistent with the results of functional verification *in vitro* by CFC assays. Notably, the combination of SR1 and LA resulted in a reconstruction level of each lineage similar to that of fresh (Figures 4D,E). Taken together, our data revealed that LA treatment led to unaffected engraftment of human cells both in PB and BM of immunodeficient mice and did not alter the capacity of the engrafted cells to retain long-term hematopoietic repopulation. Importantly, the combination of reagents such as SR1 and LA could optimize outcomes of engraftment.

### LA Enhanced the Antioxidant Activity of HSCs by Reducing ROS Levels Intracellularly and Mitochondrially

**FIGURE 5:**
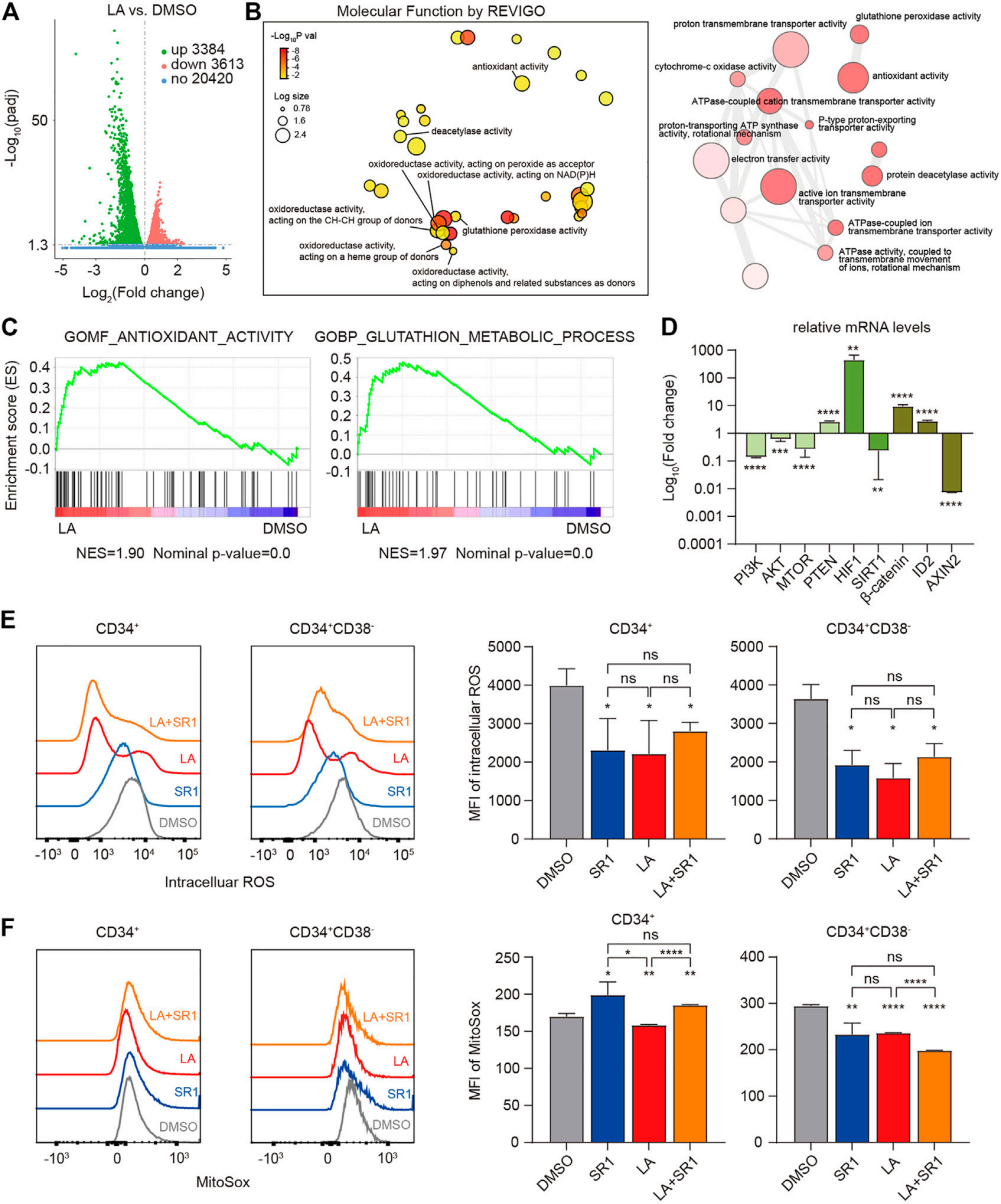
LA-induced HSCs maintenance works by enhanced antioxidant capacity. **(A,B)**, RNA-seq profiling of LA cultured UCB CD34^+^ cells. **(A)** Volcano plot of differentially expressed genes (DEGs) between LA- and DMSO-cultured UCB CD34^+^ cells (3384 up-regulated and 3613 downregulated, |log2 fold change | > 0.0, and padj <0.05). **(B)** Interactive graph of major gene ontology molecular function (GO:MF) terms analysis by REVIGO (left). Bubble color indicates -Log10 *p* value; bubble size indicates the frequency of the GO term. Highly similar GO terms are linked by edges in the graph, where the line width indicates the degree of similarity (right). **(C)** GSEA enrichment plots of the gene sets enriched in LA vs. DMSO. Normalized enrichment score (NES) is shown. **(D)** Relative expression of metabolism-related and stemness-related genes using real-time PCR. Log_10_ of the fold change of mRNA levels of LA/DMSO is shown (*n* = 3). GAPDH served as a loading control. **(E,F)** Intracellular ROS levels **(E)** and mitochondrial ROS levels **(F)** of CD34^+^ and CD34^+^CD38^−^ subpopulations in UCB CD34^+^ cells after a serum-based 7-d culture with DMSO, SR1, LA ,or LA + SR1 (*n* = 3). All data represent the means ± SD. Compared with DMSO unless specified. *p < 0.05, **p < 0.01, ***p < 0.001, ****p < 0.0001 and ns = not significant by two-tailed unpaired Students’ *t*-test.

To elucidate the molecular mechanism by which LA expands HSCs, RNA-sequencing (RNA-seq) of the *in vitro* human UCB-CD34^+^ cells was performed to obtain gene expression profiling in both the DMSO- and LA-treated groups, which showed that LA treatment resulted in significantly differential expression of 3384 upregulated and 3613 downregulated genes (Figure 5A). Based on these differentially expressed genes, we visualized the interactions among major gene ontology molecular function (GO:MF) terms by REVIGO, which showed that numerous genes have significantly increased oxidoreductase activity and antioxidant activity in the LA-treated group (Figure 5B), indicating that oxidation-reduction reactions might be one key focal point to explore how LA regulates HSCs’ functions. Consistent with the results of REVIGO analysis, subsequent gene set enrichment analysis (GSEA) data revealed that two sets of genes in antioxidant- and glutathione-related pathways (see in Methods) were upregulated in LA-treated UCB-CD34^+^ cells (Figure 5C). Furthermore, qRT-PCR analysis showed that the genes phosphatidylinositol 3-kinase (PI3KCA), protein kinase B (AKT3), mammalian target of rapamycin (mTOR), hypoxia-inducible factor-1 A (HIF-1A), catenin beta 1, β -catenin (CTNNB1), and inhibitor of DNA binding (ID2) were upregulated while the genes phosphate and tensin homolog (PTEN), sirtuins 1 (SIRT1), and axin two which degrades β -catenin (CTNNB1) were downregulated (Figure 5D), which suggested that the PI3K/Akt/mTOR (Mansell et al., 2021; Testa et al., 2016), SIRT1/HIF-1A (Ballen et al., 2013; Suda et al., 2011; Testa et al., 2016), and Wnt/*β*-catenin signaling pathways (Huang et al., 2012) might participate in the regulation of HSCs by LA.

Several studies have demonstrated that HSCs tend to reside in a hypoxic BM microenvironment and generate energy merely through anaerobic glycolysis by limiting the production of ROS to maintain their stemness and quiescence, which varies from their progenitor cells (Suda et al., 2011; Testa et al., 2016, Vlaski-Lafarge and Ivanovic, 2015). Therefore, we measured the ROS levels in both the cytoplasm and mitochondria of CD34^+^ and CD34^+^CD38^−^ cells from human UCB-CD34^+^ cells cultured *in vitro* with DMSO, LA, SR1, or SR1 plus LA by flow cytometry. Mean fluorescence intensity (MFI) of intracellular ROS measurement showed that SR1 treatment or LA treatment significantly decreased the ROS levels in CD34^+^ and CD34^+^CD38^−^ cells compared with that of the DMSO-treated group, while combining SR1 and LA did not decrease the ROS level further (Figure 5E). We also found that LA-treated cells had significantly lower mitochondrial ROS levels in CD34^+^ and CD34^+^CD38^−^ cells when compared with the DMSO- or SR1-treated groups (Figure 5F). In addition, mitochondrial ROS was reduced by SR1 treatment only in the CD34^+^CD38^−^ subpopulation rather than in the CD34^+^ subpopulation and combining SR1 with LA could reduce mitochondrial ROS further (Figure 5F). In summary, our experiments measuring ROS after treatment indicated that LA treatment led to significantly reduced intracellular and mitochondrial ROS levels in both CD34^+^ and CD34^+^CD38^−^ cells, and the combination treatment of SR1 with LA selectively reduced mitochondrial ROS level in CD34^+^ and CD34^+^CD38^−^ cells.

## Discussion

In this study, we identified LA, a dimeric phthalide derivative that could be a promising candidate to expand HSPCs *ex vivo*. Our data confirmed that LA treatment could not only expand phenotype-defined HSPCs *in vitro*, in particular in long-term HSCs which can be significantly expanded by LA in culture system with or without serum, but also retain their capacities of short-term and long-term colony formation and multilineage reconstitution in immunodeficient mice at the optimal dose of 10 µM. Phthalides are mainly extracted from the volatile oil of the plant Chuanxiong and with the rapid development of analytical techniques, hundreds of new phthalides have been identified and isolated in recent years (Chen et al., 2018). The superior activity of LA in proliferating HSCs suggests that nature is a rich library for storing diverse lead compounds which can be structurally modified or reorganized to achieve different bioactivities. Strikingly, we observed that monomer **8** itself exhibits no expansion effect on HSPCs, yet its dimer **2** (LA) exhibits potent activity, which suggests that the size and structure of the compound have a great influence on the potential target of LA. Moreover, although they are both dimers of compound **8**, the activity of LA (compound **2**) is totally different from that of compound **9** due to a different pattern of 4 + 2 cycloaddition. Perhaps the polymerization pattern subsequently impacts the spatial structure of the molecule, which determines its preferred binding targets and, in turn, distinct physiological reactions. If extending screening, more candidates belonging to phthalide derivatives may be identified. Additionally, it is notable that the expansion activity on HSPCs of phthalides is novel and varies from that of previously reported pharmacological properties such as anti-inflammation, analgesia, anti-thrombosis, anti-tumor, etc. (Chen et al., 2018), reminding us of the “new use of old medicine”.

Drug combination is likely to represent a key strategy for the future treatment of various diseases. Our experiments of xenotransplantation in immunodeficient mice demonstrated that UCB-CD34^+^ cells cultured with SR1 plus LA prior to transplantation exhibited slightly improved engraftment in PB and BM and a similar reconstitution level as that of the fresh UCB group. The aim of a drug combination is to achieve complementary advantages and reduce adverse effects. SR1 is reported to be mostly active on primitive normal hematopoietic progenitors and leukemia stem cells (LSCs) (Fares et al., 2015), while our data showed that LA had superior activity on long-term HSCs. Thus, combining SR1 and LA could make up for the deficiency of LA in expanding HPCs to fulfill complete proliferation of UCB-derived cells, even if the combination of LA and SR1 did not achieve expected combined effect on some type of colony and engraftment capacity, which is consistent with previous studies that show synergistic effect of the combination of SR1 and UM171 merely restricted to progenitor cell subpopulation (Zimran et al., 2021). This phenomenon reminds us that a combination of various agents with stem cell activity may lead to senescence or simply prove redundant. Essentially, drug combinations in this context refers to simultaneous activation of several signaling pathways related to hematopoiesis to gain ideal expansion outcomes. Previous studies have provided distinct mechanisms whereby small molecules expand *ex vivo* UCB-CD34^+^ cells, taking SR1 as an example, which serves as an aryl hydrocarbon receptor (AhR) antagonist (Boitano et al., 2010); this has extended our understanding of hematopoiesis and the mechanisms involved. Additionally, increasing the number of investigations in the biology of HSCs will provide strong theoretical foundations for this work. Notably, this area of elucidation is not limited only to a combination between small molecules, but also extends to a combination among small molecules, cytokines, and three-dimensional microcarriers exemplified by zwitterionic hydrogel (ZTG) (Bai et al., 2019), which has been regarded as a novel efficient culture system for HSCs’ expansion and merits further consideration.

Through our research into the mechanisms underlying our observations, we primarily revealed that LA treatment led to inhibition of the PI3K/Akt/mTOR signaling pathway, which plays a crucial role in regulating metabolism through complex mechanisms. For example, activation of mTORC1 by Akt and upregulation of HIF1 promote glycolysis by converting pyruvate to ATP molecules and lactate (Nepstad et al., 2020). Transferring to glycolysis means less NAD^+^, a deacetylated substrate of SIRT1, will be generated. A lower NAD^+^ level cannot make SIRT1 better for deacetylation (Hwang and Song, 2017). On the contrary, upregulated HIF1A combines with downregulated SIRT1 which in turn acetylates and activates HIF1A. Different substrates that SIRT1 binds to determine different biological functions it exerts. Activation by downregulated SIRT1 is critical for HIF1A to function because upregulated SIRT1 leads to the deacetylation and inactivation of HIF1A even in hypoxia (Lim et al., 2010). This is the reason why some studies found that loss of SIRT1 leads to elevated ROS and DNA damage in mice (Bigarella et al., 2014). In contrast to HIF2A and HIF3A, HIF1A remains stable and promotes transcription of genes in the nucleus under hypoxic conditions to attenuate high ROS levels and severe DNA damage in cells (Bigarella et al., 2014; Lim et al., 2010; Testa et al., 2016). A number of studies have indicated that HIF activation can induce HSCs both *in vitro* and *in vivo* and potentiate their self-renewal potential (Forristal et al., 2013; Nombela-Arrieta et al., 2013), which may be because HIF1A drives cellular metabolism toward anaerobic glycolysis instead of mitochondrial respiration (Papa et al., 2019). However, the specific molecular mechanism whereby LA regulates the biology of HSCs through HIF1A deserves further study, including the reason why HIF1A was upregulated and SIR1 was downregulated after LA treatment. Additionally, the Wnt/*β*-catenin signaling pathway was activated, which could partially account for maintenance of HSCs (Huang et al., 2012).

In conclusion, we identified a phthalide derivative dimer, LA, which can expand HSPCs *in vitro* and maintain unaffected homing or engraftment of human HSCs cultured with LA *in vivo* with functional long-term hematopoietic reconstitution capability. Based on the relationship between hematopoiesis and redox status, we found that LA maintained quiescence and stemness of HSCs via terminal SIRT1/HIF1 collaboration to reduce intracellular and mitochondrial ROS level.

## Data Availability

The datasets presented in this study can be found in online repositories. The names of the repository/repositories and accession number(s) can be found in the article/Supplementary Material.
