# Supplementary material for: Levistilide A Promotes Expansion of Human Umbilical Cord Blood Hematopoietic Stem Cells by Enhancing Antioxidant Activity
Source: Front Pharmacol. 2022 Feb 17;13:806837. doi: 10.3389/fphar.2022.806837 (PMC8895481; doi:10.3389/fphar.2022.806837)
Supplement: Supplementary file 1 [file DataSheet1.DOCX]

Supplementary Material

# NMR SPECTRA (^1^H NMR, ^13^C NMR) OF CPMPOUNDS 1 AND 2, HRMS COPIES OF COMPOUNDS 1 AND 2, HPLC ASSESSMENT OF PURITY FOR COMPOUNDS 1 AND 2

**Compound 1**


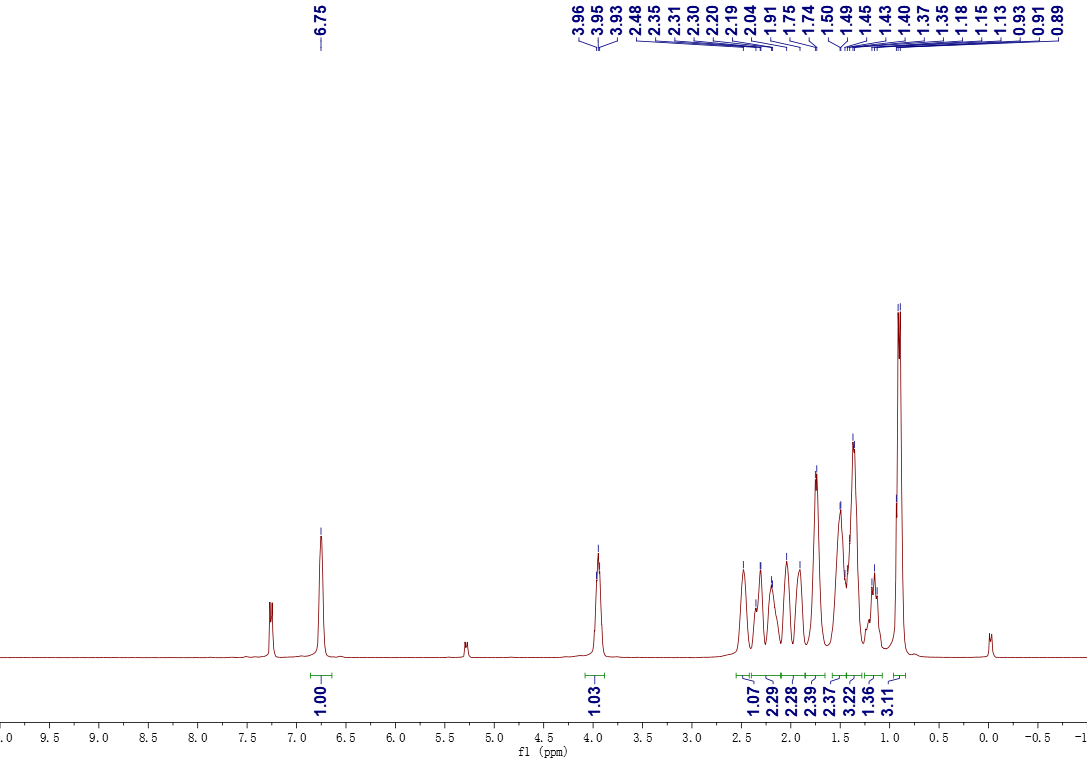


^1^H NMR copy of compound **1**


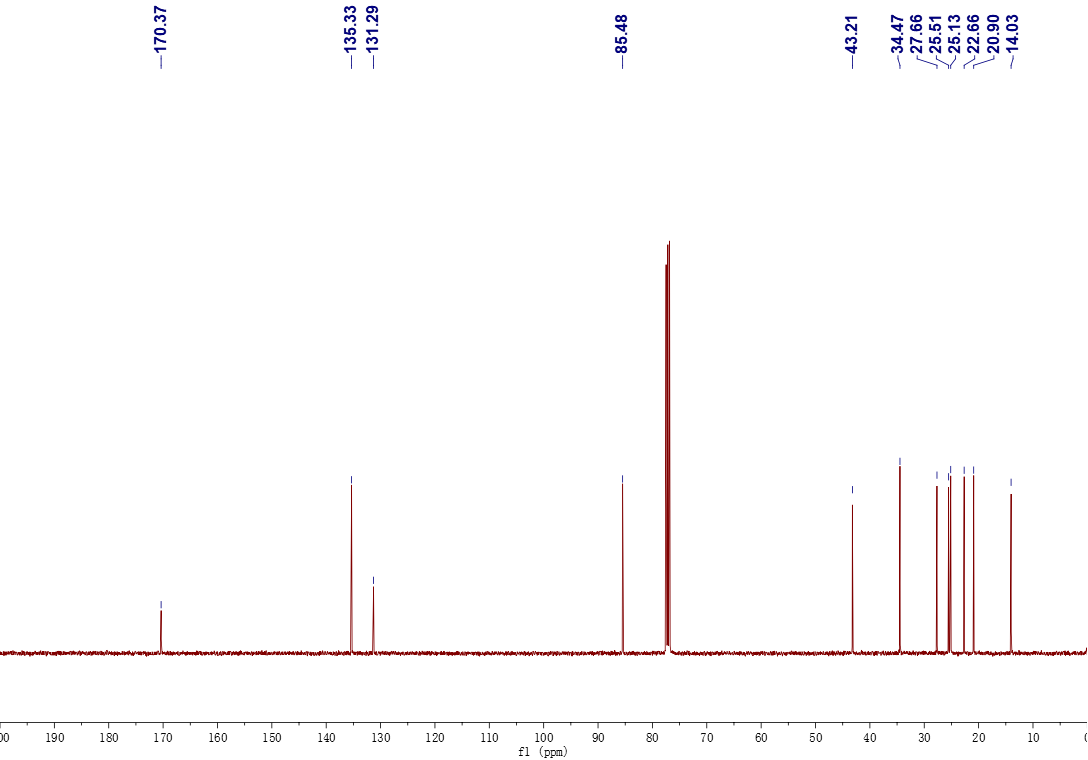


^13^C NMR copy of compound **1**


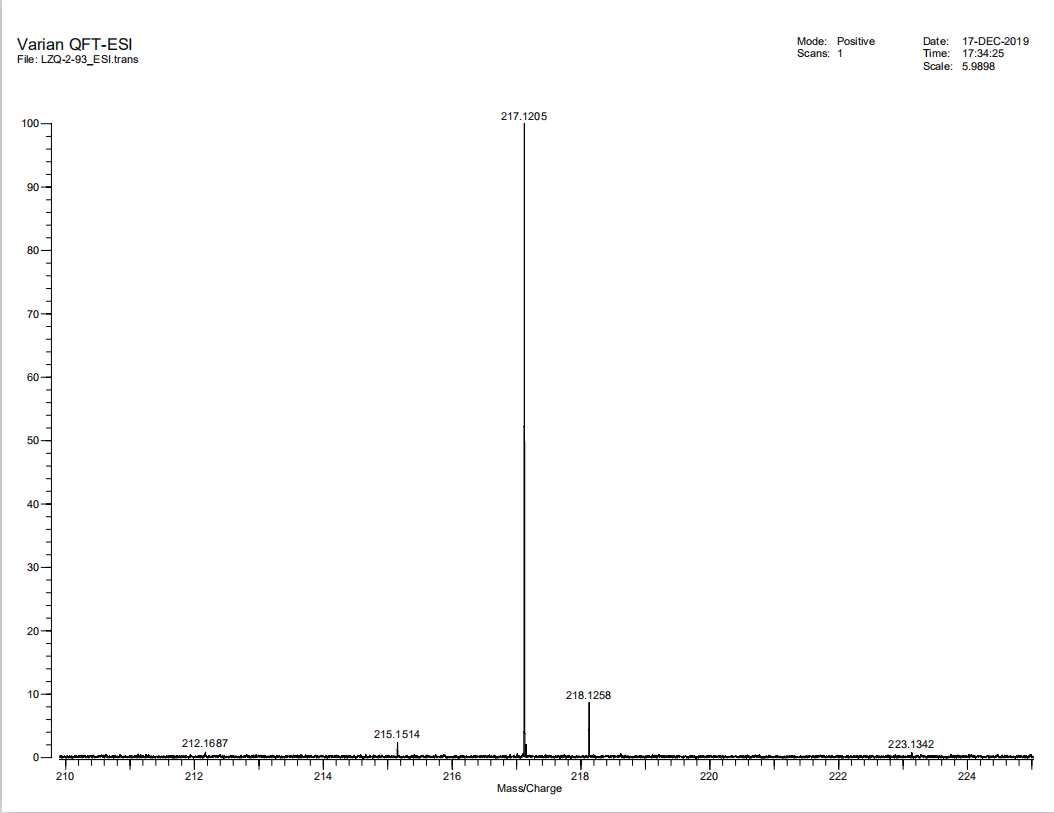


HRMS (ESI) copy of compound **1**


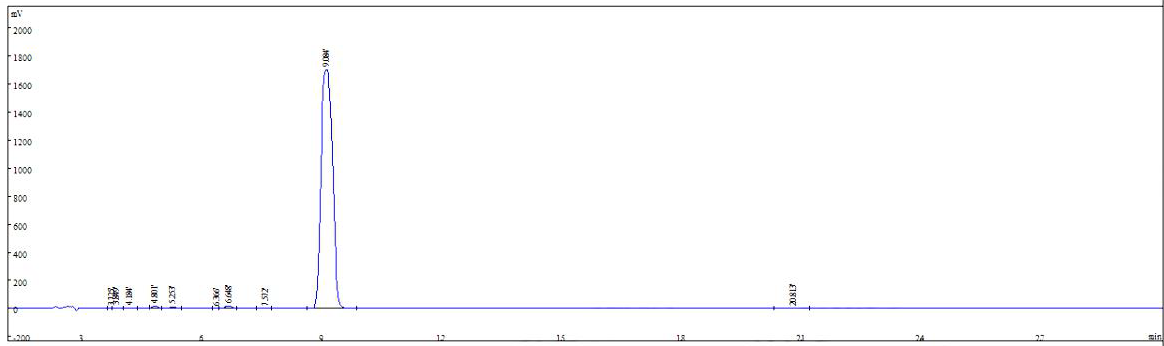


HPLC analysis of compound **1**

**Compound 2**


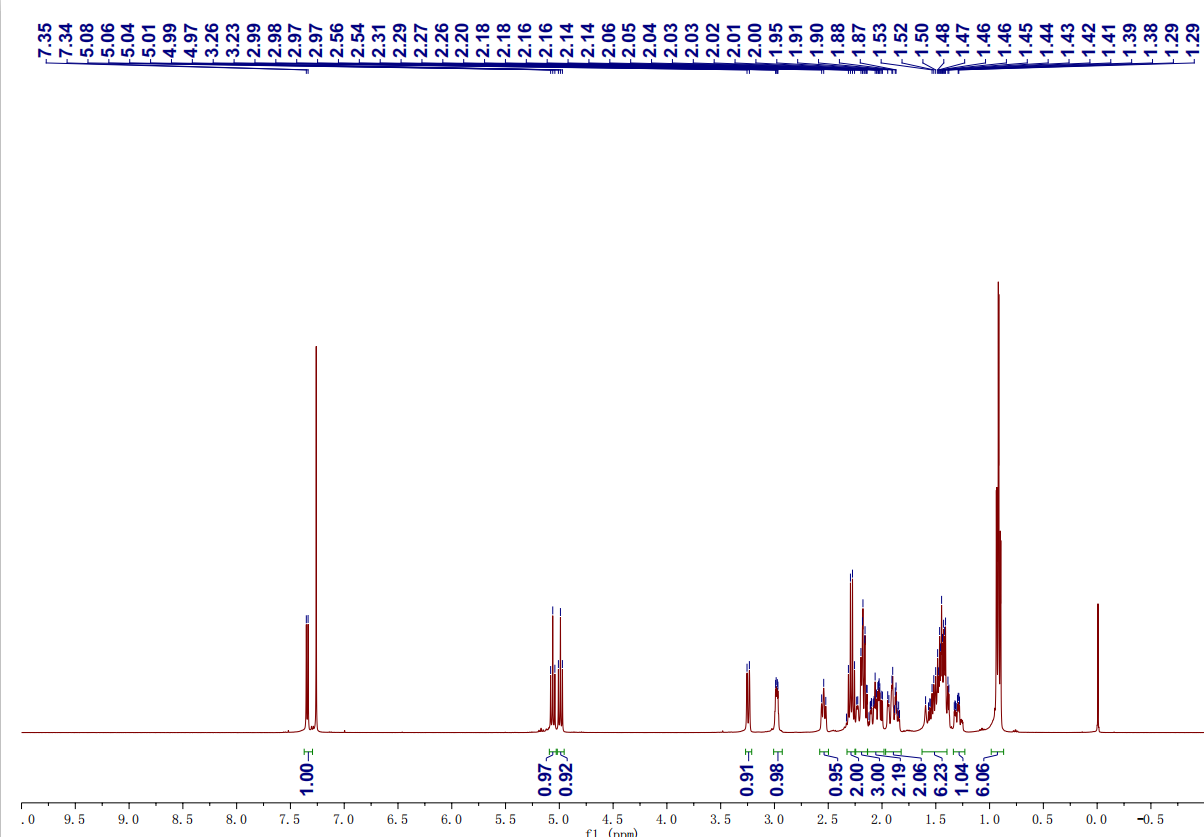


^1^H NMR copy of compound **2**


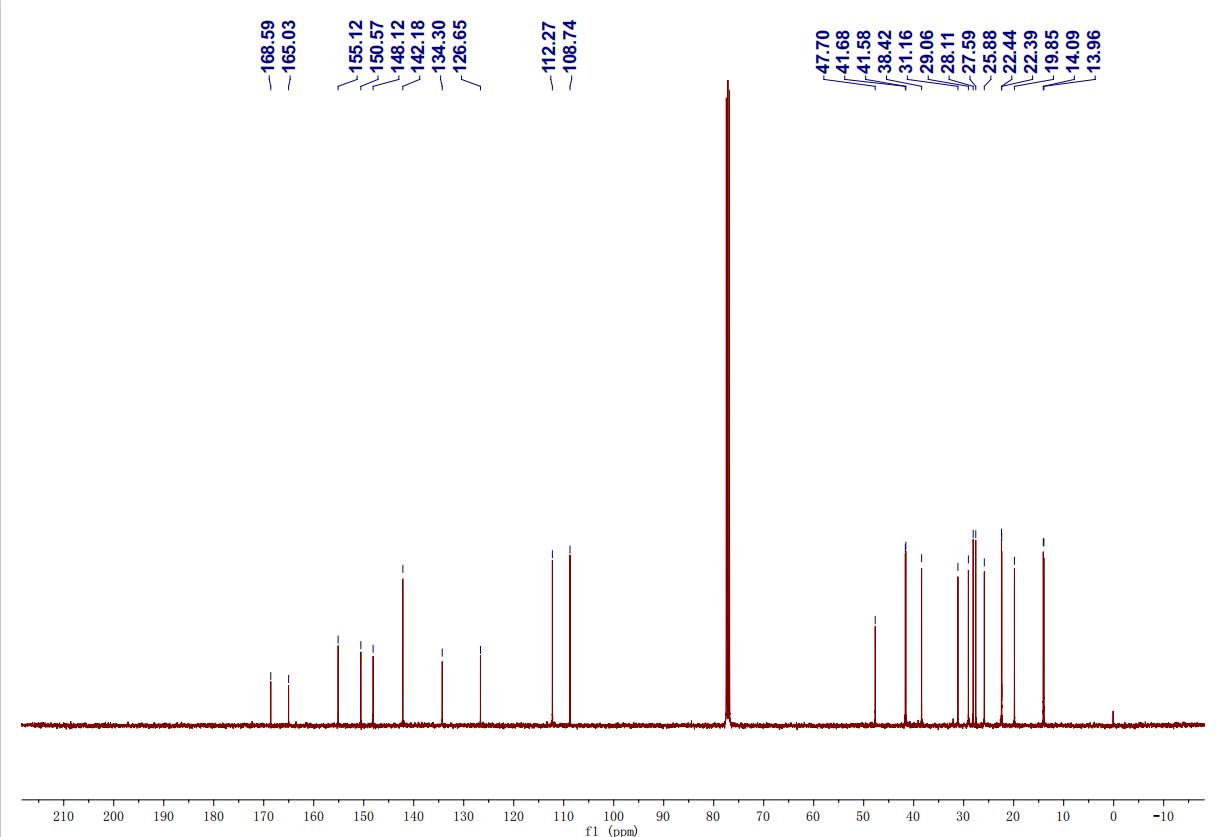


^13^C NMR copy of compound **2**


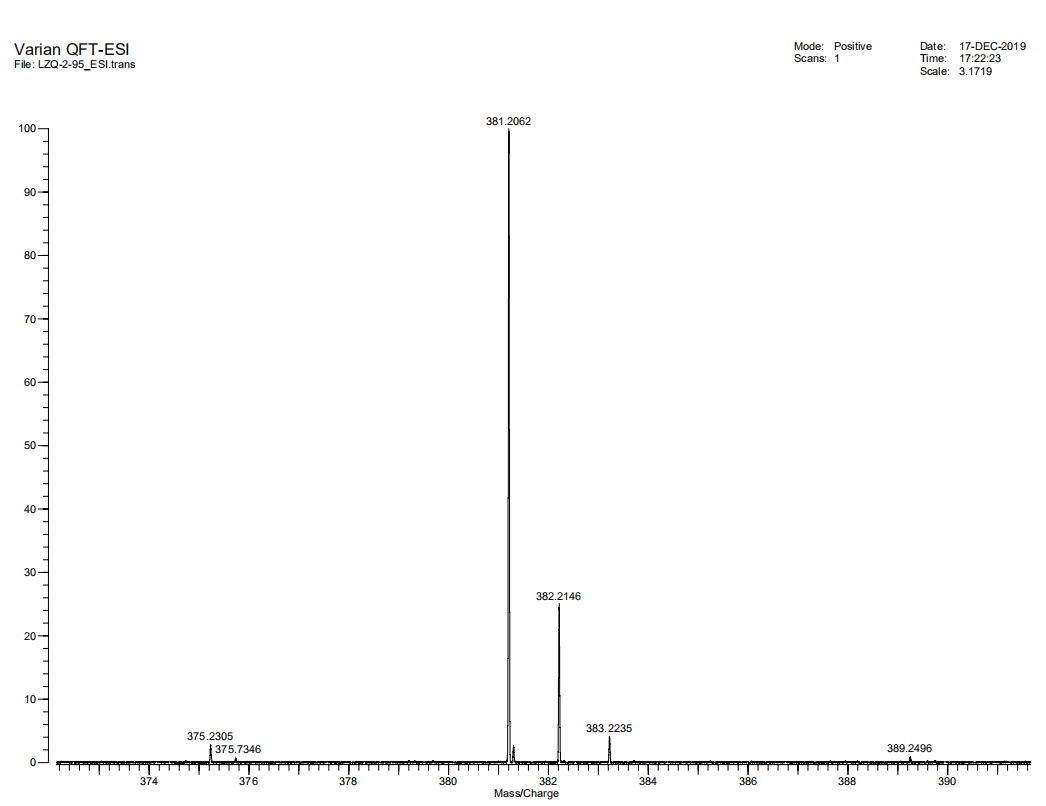


HRMS (ESI) copy of compound **2**

**
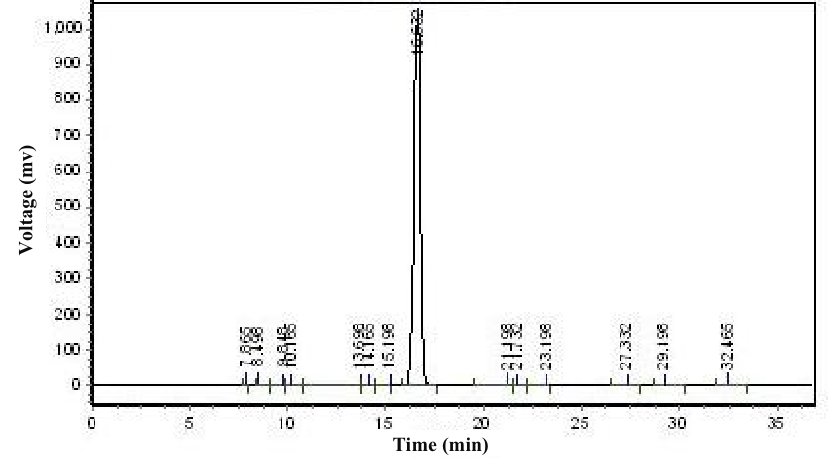
**

HPLC analysis of compound **2**

**Compound 3**

^1^H NMR and ^13^C NMR Copies of compound **3**

**Compound 4**

^1^H NMR and ^13^C NMR Copies of compound **4**

**Compound 5**

^1^H NMR and ^13^C NMR Copies of compound **5**

**Compound 6**

^1^H NMR and ^13^C NMR Copies of compound **6**

**Compound 7**

^1^H NMR and ^13^C NMR Copies of compound **7**

**Compound 8**

^1^H NMR and ^13^C NMR Copies of compound **8**

**Compound 9**

^1^H NMR and ^13^C NMR Copies of compound **9**

# PRIMER SEQUENCE OF TARGET GENES

| **Primers** | **Sequences** | **Base number** |
| --- | --- | --- |
| PI3KA U | GAGTACCTTGTTCCAATCCCAG | 22 |
| PI3KA D | TTCCTCTTTAGCACCCTTTCG | 21 |
| AKT3 U | TTTTCTCTATTATTTGGGCTGAGTC | 25 |
| AKT3 D | CCCCTCTTCTGAACCCAACC | 20 |
| MTOR U | TCATTCACCAGCTTCTCACAG | 21 |
| MTOR D | CACCTCAAACATGCCTTTCAC | 21 |
| PTEN U | AGTCCAGAGCCATTTCCATC | 20 |
| PTEN D | TTCCTGTATACGCCTTCAAGTC | 22 |
| HIF1A U | GGAAACTCAAAACCTGAAGAATTGG | 25 |
| HIF1A D | TCAATATCCAAATCACCAGCATCC | 24 |
| SIRT1 U | TCAGGTCAAGGGATGGTATTTATG | 24 |
| SIRT1 D | TGTTCCAGCGTGTCTATGTTC | 21 |
| CTNNB1 U | TTGTGCGTACTGTCCTTCG | 19 |
| CTNNB1 D | AAGCAACTGAACTAGTCGTGG | 21 |
| ID2 U | CATCCCACTATTGTCAGCCTG | 21 |
| ID2 D | AGGTCCATTCAACTTGTCCTC | 21 |
| AXIN2 U | GGATCACTGGCTCCGCGA | 18 |
| AXIN2 D | GCTCATCTGAACCTCCTCTCTTT | 23 |
